# Transnasal Endoscopic Orbital Decompression Combined with an Enhanced Recovery after Surgery Protocol in Graves Ophthalmopathy

**DOI:** 10.1155/2022/6382429

**Published:** 2022-09-16

**Authors:** Hui-Qing Xu, Qian Wang, Li-Li Hu, Hong Li, Feng Peng

**Affiliations:** Otolaryngology-Head and Neck Surgery, The Third Affiliated Hospital, Sun Yat-Sen University, Guangzhou, Guangdong 510630, China

## Abstract

**Objective:**

Although enhanced recovery after surgery (ERAS) was shown to improve patients' recovery after surgery and transnasal endoscopic orbital decompression has been associated with lesser risks of postoperative complications compared to other surgical techniques in treating Graves ophthalmopathy (GO), there are currently no clinical studies on the application of ERAS in transnasal endoscopic orbital decompression. This study aimed to investigate the potential effects of combining transnasal endoscopic orbital decompression with ERAS in the treatment of GO.

**Methods:**

A retrospective analysis was performed for 5 GO patients (10 eyes) treated with transnasal endoscopic orbital decompression from January 2021 to December 2021 at the Third Affiliated Hospital of Sun Yat-Sen University. All patients underwent ERAS, and the effects of ERAS on the postoperative complications and recovery of patients were evaluated.

**Results:**

Ophthalmological examination showed that GO patients had good correction of exophthalmos after surgery combined with ERAS. Specifically, the exophthalmos reduction in subjects was 0.9–2.1 mm, with a mean reduction of 1.23 mm. In addition, a visual acuity improvement of 0.15–0.4, with an average improvement of 0.23, was also observed. Further, the Scale of Quality of Life for Diseases with Visual Impairment (SQOL-DVI) showed that, compared with before surgery, the patients' QOL was significantly improved 2 weeks after surgery. Before surgery, there were 2 patients with diplopia and blurred vision, and after postoperative adaptive exercise, the symptoms of these 2 patients disappeared after 6 months of follow-up. As for the other 3 patients, they had no diplopia or blurred vision after surgery.

**Conclusion:**

This observational study found that transnasal endoscopic orbital decompression might be effective in treating GO, and ERAS might be considered an important adjunct to improving perioperative care and postoperative recovery.

## 1. Introduction

Graves ophthalmopathy (GO), also called thyroid-associated ophthalmopathy, is an autoimmune and potentially sight-threatening disease [1]. It was reported to affect 25%–50% of patients with hyperthyroidism secondary to Graves' disease and 2% of patients with chronic thyroiditis [2]. Most patients with GO are accompanied by hyperthyroidism, exophthalmos, diplopia, different degrees of visual loss, and might even have exposed corneal ulcers. These symptoms may seriously affect the patients' appearance and quality of life (QOL) and increase their medical expenditure.

Treatments of GO include high-dose corticosteroids, retrobulbar radiotherapy, immunosuppressive agents, and orbital decompression [3]. Systemic steroids are usually required for months at high doses but have undesirable adverse events. In addition, the signs and symptoms may return when the medication is tapered [4]. Although external beam radiotherapy might be effective in the acute phase of the disease, it cannot significantly improve proptosis. Thus, several alternative decompressive techniques and approaches have been proposed since 1911, when Dollinger first described surgical orbital decompression [5]. Orbital decompression can be performed in patients with severe symptoms affecting QOL and poor response to nonsurgical treatment and has been associated with improvements in proptosis, corneal exposure, and orbital neuropathy [6–8]. In addition, due to advantages such as avoiding external ocular scars as they can be performed via a transnasal route under precise endoscopic guidance, providing clear surgical fields, and being cosmetically more acceptable, transnasal endoscopic surgery is increasingly used in clinical practice [9]. Thus, although high-dose corticosteroid therapy remains the first-line management for GO, in case of treatment failure, surgical orbital decompression is a definite choice.

However, despite the many advantages, a purely endoscopic approach may also have several limitations, such as difficulty in removing the bone lateral to the inferior orbital nerve or anterior to the middle meatal antrostomy, and decompression of the medial wall and floor only may result in significant medial orbital prolapse, leading to diplopia [10]. In this regard, enhanced recovery after surgery (ERAS) refers to a series of modified measures applied during the perioperative period to accelerate postoperative recovery and was first introduced 15 years ago for colorectal surgery [11]. Specifically, ERAS can accelerate recovery, maintain body composition, and shorten discharge time without affecting morbidity by combining multimodality pathways such as anesthesia, surgery, nursing, and perioperative management. ERAS protocols are widely used in some specialty areas, including urology, vascular and plastic surgery [12–14]. However, there are currently no clinical studies on the application of ERAS in transnasal endoscopic orbital decompression.

To tackle these existing limitations in the current literature, we undertook this observational study to describe our experience with the potential use of transnasal endoscopic orbital decompression with ERAS in treating GO and to provide preliminary medical evidence for the potential application of ERAS in this setting.

## 2. Data and Methods

### 2.1. Selection of the Study Subjects

The data of GO (10 eyes) cases who received transnasal endoscopic orbital decompression and ERAS nursing intervention at our hospital from January 2021 to December 2021 were retrieved and assessed. The study inclusion criteria included (1) patients who met the diagnostic criteria for GO [15]. On the basis of typical clinical manifestations/abnormalities in thyroid function tests, ocular computed tomography/magnetic resonance imaging (CT/MRI) revealed significantly increased fat in the orbit, exophthalmos, and intraocular muscle hypertrophy; (2) After treatment, the patient's condition was well controlled for at least 6 months, but there were still GO-related exophthalmos and decreased visual acuity; (3) the patients could communicate normally and cooperate with treatment, and; (4) the patients and their families were aware of the treatment protocol and provided signed informed consent form for the anonymous use of their data for research purposes. Cases were excluded if (1) the patient could not receive general anesthesia due to diseases like severe liver, kidney, and cardiopulmonary dysfunctions; (2) suffered from severe chronic sinusitis and nasal polyps, as well as a failure in inflammation control by drug therapy; (3) could not communicate normally or suffer from unstable mental disorders, and; (4) had unrealistic surgical outcome expectations. After data selection, the clinical information of the enrolled subjects was analyzed. The study protocol was approved by the Medical Ethics Review Board of the Third Affiliated Hospital of Sun Yat-Sen University (2020-02-001-01), and all patients signed informed consent forms.

### 2.2. Surgical Methods and Postoperative Treatment

All patients underwent nasal endoscopic bilateral orbital decompression under general anesthesia. Briefly, the ethmoid and maxillary sinuses were opened via conventional methods under a nasal endoscope to remove the orbit's medial wall and inferior wall bone. Next, a transverse incision was made on and below the medial rectus muscle to incise the orbital fascia, and then the orbital fat was aspirated to the greatest extent with suction forceps. Two patients (4 eyes) were given endoscopic optic nerve decompression due to a short-term sharp decrease in their visual acuity. Specifically, the optic canal was located on the lateral wall of the ethmoid and sphenoid sinus. Then, 1/2 perimeter of the optic canal bone was abraded, followed by optic nerve decompression. Absorbable material “NasoPore” and macromolecule expansive sponge were used as hemostasis packing in the nasal cavity. After surgery, the patients were given methylprednisolone sodium succinate (methylprednisolone, 1 g QD) hormone pulse therapy for 5 days continuously. In addition, an intravenous drip using broad-spectrum antibiotics was performed for 5 d. Additionally, the sponge in the nasal cavity was removed 48 hours after surgery.

### 2.3. Postoperative Care

#### 2.3.1. Enhanced Recovery after Surgery

The ERAS-MDT team is composed of otorhinolaryngologists and nurses, anesthesiologists, operating room nurses, mental health specialist nurses, dietitians, and other relevant personnel. In addition, a series of ERAS measures were clustered and implemented to promote postoperative rehabilitation. Before surgery: (1) the perioperative diagnosis and treatment process and nursing-related matters were introduced in the form of multimedia, brochures, display board, and other educational activities to obtain the cooperation of patients and help patients build a reasonable expectation for the prognosis; (2) according to a survey scale, targeted psychological counseling was performed to reduce the anxiety of patients and other negative emotions; (3) the patients were required to quit smoking and alcohol for 4 weeks; (4) diet requirements included: fasting from solid food 8 h before surgery and no water consumption 2 h before surgery. Of note, vitamin drinks were allowed 2 h before surgery at 5 mL/kg body weight; (5) oral nonsteroidal antiinflammatory drugs, such as loxoprofen sodium tablets, were administered the night before surgery for prophylactic analgesia, and; (6) antibiotics were used 30 min before surgery, and if the surgery was longer than 3 h, another dose of antibiotics was given to prevent infection. Overall, the surgery in this study was completed within 1 h. During the surgery: (1) according to the results of the Predictors score scale, a scale evaluating hypothermia risk, patients at risk of hypothermia were warmed with a warming blanket 15 min before anesthesia induction to ensure their body temperature was ≥36.0°C (2) the patients were required to manage body fluids well, reduce the input of fluid and sodium salt fluid, and appropriately reduce the intake of crystalloid fluid, and; (3) the circulatory monitoring of patients was improved. As for postoperative care, (1) 2 h after returning to the ward and following an assessment by the nurse, warm and soft food was allowed orally. According to the gastrointestinal tolerance of the patient, a complete diet was allowed 4 h after surgery; (2) flurbiprofen axetil injection (50 mg) was given intravenously 2 h after surgery, and 50 mg intravenous injection was performed again at night before bedtime for preventive analgesia. Moreover, the analgesia was achieved by adding flurbiprofen axetil injection (50 mg) before the removal of packing at 48 h after surgery, so patients could pass through the perioperative period painlessly and improve their treatment experience; (3) antibiotic guidelines were strictly followed after surgery; (4) aerosol inhalation was performed twice a day to promote the discharge of secretion 2 h after returning to the ward, and; (5) according to the condition of the patients, after eating on the day following surgery, they were guided to conduct early ambulation, and activity plans were developed and implemented.

### 2.4. Discharge and Follow-Up

The patients were discharged 4–6 days after surgery and told to continue oral medication, eye drops, and eye function exercises as prescribed by the doctor. The medical staff of our department developed a personalized follow-up plan for the discharged patients and provided counseling for the patients' psychological care, nutrition, and sinonasal care. Patients were told to receive outpatient reexamination 2 weeks after discharge. Outpatient reexaminations were performed at the endocrinology and ophthalmology departments. During the reexamination, the postoperative rehabilitation and the occurrence of complications were evaluated. In addition, doctors were required to take prompt action in case of suspected complications or other recovery-related issues. After discharge, the patients were followed up by telephone every month. After surgery, the overall condition of the patients was assessed, and nursing was provided until the patients recovered stably.

### 2.5. Statistical Method

Data analysis was performed using the SPSS 20.0 software. Experimental data are presented as mean ± standard deviation (SD) and statistically analyzed using the *t*-test. *P* < 0.05 indicated statistically significant differences.

## 3. Results

### 3.1. General Clinical Information

A total of 5 GO patients aged 29–70 (47.5 ± 5.8) years were admitted from January 2021 to December 2021. They comprised 2 males and 3 females. Specifically, their disease duration was 5–120 months, with a mean duration of 30.0 ± 2.2 months. The degree of left eye exophthalmos was 23–25.7 mm, with a mean degree of 24.46 ± 1.02 mm. The degree of right eye exophthalmos was 22.5–26 mm, with a mean degree of 24.1 ± 1.25 mm. Besides, visual acuity in the left eye ranged from 0.2 to 0.8, with an average of 0.40 ± 0.35, while that of the right eye ranged from 0.25 to 1.0, with an average of 0.47 ± 0.31. Notably, there were 2 cases of diplopia and blurred vision.

### 3.2. Assessments for Quality of Life

2 weeks after surgery, the ocular symptoms, visual function, physical function, social activities, and mental health of the patients were significantly improved compared with those before surgery, and the differences were statistically significant (*P* < 0.05) (Table 1).

### 3.3. Ocular Symptoms and Visual Functions

Significant improvement was observed in exophthalmos and visual acuity after surgery. Specifically, after surgery, the exophthalmos reduction was 0.9–2.1 mm, with an average of 1.23 mm. In addition, visual acuity improvement was 0.15–0.4, with an average improvement of 0.23. Significant differences were observed in exophthalmos and right eye visual acuity before and after surgery (*P* < 0.05). However, no significant difference in left eye visual acuity score before and after surgery was observed (*P* > 0.05) (Table 2).

### 3.4. Complications after Surgery

Of the 5 investigated patients, 2 presented with diplopia and blurred vision before surgery. However, after postoperative adaptive exercise, their symptoms disappeared after 6 months of follow-up. There was no severe vascular injury, orbital hematoma, infection, cerebrospinal fluid rhinorrhea, and other conditions during the surgery.

## 4. Discussion

In this observational retrospective study, we assessed the general ocular clinical data and postoperative QOL, ocular symptoms, visual functions, and complications of GO patients who underwent transnasal endoscopic orbital decompression together with ERAS. Overall, the results showed that the treatment protocol was feasible and safe, with significant improvement in the patients' ocular symptoms, visual function, physical function, social activities, and mental health, compared with before surgery. In addition, we also observed that the preoperative diplopia and blurred vision symptoms of 2 patients were cured after the treatment provided at 6 months of follow-up.

The first report on endoscopic orbital decompression achieved a reduced axial proptosis by a mean of 5.7 mm when combined with lateral orbitotomy and 4.7 mm when decompressed by an endoscopic approach alone [16]. However, only two patients (three orbits) were purely treated with endoscopic decompression. As the techniques started to evolve, the reported mean reductions in proptosis began to improve. Wee et al. reported a series of 10 endoscopic orbital decompressions, demonstrating an average improvement of 4.4 mm (range, 3.0 mm to 4.7 mm) in orbital proptosis [17]. They also observed an improvement in visual acuity in all patients with visual impairment and recommended endoscopic orbital decompression as an alternative to traditional decompression techniques. She et al. reported a reduction in proptosis by an average of 1.93 ± 0.25 (*P* < 0.01) after 1 month and 2.07 ± 0.29 (*P* < 0.01) after 3 months postoperatively [6]. Kochetkov et al. [18] investigated the efficacy of endoscopic orbital decompression surgery for treating patients with GO and reported that the exophthalmos symptoms were reduced and visual acuity was further reduced and improved in postoperative patients with inactive GO or GO complicated with optic neuropathy. Gras-Cabrerizo et al. [19] reported that endoscopic orbital decompression significantly improved visual acuity and exophthalmos symptoms in patients with different degrees of GO. In this study, the exophthalmos reduction in patients was 0.9–2.1 mm, with an average reduction of 1.23 mm. The visual acuity improvement was 0.15–0.4, with an average improvement of 0.23, and there were significant differences in exophthalmos and the visual acuity of the right eye before and after surgery. The lower mean reduction of our study compared with the previous studies might be due to the shorter duration of assessment (2 weeks in this present study, while at least 1 month in most studies).

CSF leak is a relatively common postoperative complication observed in transnasal endoscopic procedures. Its extension of Postoperative CSF leak may range from minor subcutaneous CSF collection resolving without any treatment, puncture of subcutaneous collections, asymptomatic CSF collections in postoperative imaging, pseudo meningocele, or overt leakage from the surgical site or through the nose or ear [20]. In the study of She et al., the authors reported one case of CSF leak during their initial phase of starting transnasal endoscopic orbital decompression and highlighted the importance of an experienced endoscopist for decompression surgery [6]. In our analysis, no patients developed a severe vascular injury, orbital hematoma, infection, cerebrospinal fluid rhinorrhea, and other conditions during the surgery.

Some controversy exists regarding the incidence of postoperative diplopia and the worsening of preexisting diplopia [21]. This might be related to the considerable restriction of eye movement in all directions in a swollen orbit. When the external restriction of proptosis is relieved by decompression, the less affected muscles can function while the more swollen and fibrotic muscles are not, resulting in an asymmetric limitation of eye movement instead of the preexisting symmetric limitation [22]. In our series, none of the patients demonstrated aggravated or postoperative diplopia. In fact, there were 2 patients with diplopia and blurred vision before surgery, and after postoperative adaptive exercise, the symptoms of these two patients disappeared within 6 months of follow-up. Our outcomes were consistent with the findings of previous studies [21, 23].

To a certain extent, we believe that the absence of postoperative complications and improvement of diplopia might be related to the implementation of ERAS, which is used to improve perioperative treatment protocols to help patients reduce the psychological and physical traumatic stress response, promoting rapid recovery, shortening the length of hospital stay and improving their quality of life. In China, Song first proposed the implementation of ERAS in the department of otorhinolaryngology in 2017 [24]. Also, studies by Wu et al. and Qi et al. [25–27] have demonstrated that ERAS helped patients undergoing endoscopic sinus surgery to relieve pain faster, reduce anxiety, ameliorate sleep quality, alleviate anesthesia-induced adverse reactions such as nausea and vomiting, promote postoperative rehabilitation, and improve the quality of life after surgery. In this study, the Scale of Quality of Life for Diseases with Visual Impairment (SQOL-DVI) proposed by ophthalmologists was applied to assess the life and psychological status of patients [28], mainly including ocular symptoms, visual function, physical function, social activities, and mental health. The assessment outcomes revealed a lower score of SQOL-DVI before surgery than after surgery. Moreover, postoperative patients had stable mental and emotional health and could cooperate reasonably with the treatment. Based on our current literature search, this study is the first to implement the ERAS protocol with transnasal endoscopic orbital decompression in treating GO patients. Based on previous literature regarding the efficacies of ERAS in the surgical treatment of other diseases, we believe that the multidisciplinary ERAS team of this study was able to timely deal with any issues the patients had during their recovery, which might have helped avoid complications and provided proper lifestyle guidance and habit modification for improved recovery.

Despite the interesting findings reported here, there were some limitations worth mentioning. First, although the observations described in this study might be consistent with some previously reported larger series, the results should be cautiously considered due to the limited number of patients. Second, we could not set a control group to fully determine the efficacy of the ERAS care regimen. Thus, whether the patient's postoperative QOL, improvement in ocular symptoms, and no postoperative complications were the results of the surgery or the impact of the surgery combined with the ERAS care regimen should also be clarified in future studies. Third, a single-center small sample study lacks certain statistical inference persuasiveness, so further sample size expansion is required in the future.

## 5. Conclusion

In this study, we applied transnasal endoscopic orbital decompression combined with ERAS to treat GO patients and observed significant improvement in ocular symptoms, visual function, physical function, social activities, and mental health of the patients compared with before surgery. In addition, no postoperative complications were observed, and the preoperative diplopia of 2 patients was cured during follow-up. Thus, these results demonstrated the feasibility, safety, and potential clinical application of this treatment protocol in treating GO patients. However, considering the retrospective nature of this study, small sample size, and lack of a control cohort to confirm the impact of ERAS, larger series of patients using randomized prospective settings with well-defined study groups is needed to confirm these findings.

## Figures and Tables

**Table 1:** Comparison of SQOL-DVI scores before and after surgery in patients with Graves ophthalmopathy.

Factors	Before surgery	Two weeks after surgery	*t*	*P* value
Symptoms and visual function	54.6 ± 4.78	34.6 ± 8.51	4.623	0.01
Physical function	28.2 ± 2.28	14.6 ± 2.07	7.532	0.002
Social activities	28.6 ± 3.36	10.4 ± 3.58	7.01	0.002
Mental health	28.6 ± 1.14	13.4 ± 3.05	14.905	<0.001

*Note.* SQOL-DVI, the scale of quality of life for diseases with visual impairment.

**Table 2:** Comparison of ocular symptoms and visual function before and after surgery in patients with Graves ophthalmopathy.

Factors	Left eyes		Right eyes	
	Exophthalmos	Visual acuity	Exophthalmos	Visual acuity
Before surgery	24.46 ± 1.02	0.40 ± 0.35	24.1 ± 1.25	0.47 ± 0.31
Two weeks after surgery	23.3 ± 0.84	0.56 ± 0.27	22.98 ± 1.00	0.70 ± 0.24
*t*	8.506	−1.969	9.025	−2.994
*P*	0.001	0.12	0.001	0.04

## Data Availability

The data used to support the findings of this study are available from the corresponding author upon request.
